# Strategies to strengthen the provision of mental health care at the primary care setting: An Evidence Map

**DOI:** 10.1371/journal.pone.0222162

**Published:** 2019-09-06

**Authors:** Witness Mapanga, Daleen Casteleijn, Carmel Ramiah, Willem Odendaal, Zolani Metu, Lesley Robertson, Jane Goudge

**Affiliations:** 1 Centre for Health Policy, School of Public Health, University of the Witwatersrand, Johannesburg, South Africa; 2 Occupational Therapy Department, Faculty of Health Sciences, University of the Witwatersrand, Johannesburg, South Africa; 3 Sedibeng District, Gauteng Department of Health, Johannesburg, South Africa; 4 Epidemiology and Biostatistics Division, School of Public Health, University of the Witwatersrand, Johannesburg, South Africa; 5 Health Systems Research Unit, South African Medical Research Council, Cape Town, South Africa; 6 Department of Psychiatry, Stellenbosch University, Cape Town, South Africa; 7 Department of Psychiatry, School of Clinical Medicine, University of the Witwatersrand, Johannesburg, South Africa; University of Ghana College of Health Sciences, GHANA

## Abstract

In a deinstitutionalised mental health care system, those with mental illness require complex, multidisciplinary and intersectoral care at the primary or community service setting. This paper describes an Evidence Map of different strategies to strengthen the provision of mental health care at the primary health care (PHC) setting, the quality of the evidence, and knowledge gaps. Electronic and reference searching yielded 2666 articles of which 306 qualified for data extraction. A systematic review methodology identified nine different strategies that strengthen the provision of mental healthcare and these strategies are mapped in line with the outcomes they affect. The top three strategies that were reported the most, included strategies to empower families, carers and patients; integration of care or collaborative interventions; and e-health interventions. The least reported strategy was task shifting. The Evidence Map further shows the amount and quality of evidence supporting each of the listed strategies, and this helps to inform policy design and research priorities around mental health. This is the first systematic Evidence Map to show the different strategies that strengthen the provision of mental healthcare at PHC setting and the impact these strategies have on patient, hospital and societal level indicators.

## Introduction

Mental illness is a major contributor to disability, morbidity and mortality globally [1]. The World Health Organisation (WHO) defines mental health as "a state of well-being in which every individual realizes his or her potential, can cope with the normal stresses of life, can work productively and fruitfully, and can make a contribution to her or his community" [2]. A systematic analysis for the Global Burden of Disease study had five types of mental illnesses, being anxiety disorders, major depression, schizophrenia, bipolar disorder and dysthymia, in the top 20 global disease burden [3]. In addition, evidence indicates that these global estimates are likely to be underestimating the true burden of mental illness [3]. Quantifying the burden of mental illness is challenging because of difficulties with definitions, the overlap of mental illness with neurological conditions, and the neglect and stigma people with mental illness experience [4–5]. After adjusting for some of the reported reasons that skewed the mental illness disease burden, Vigo et al reported that mental illness accounts for 9.8% of disability-adjusted life years (DALYs), and 32.4% of years lived with disability (YLDs) globally [6].

Despite such a high global disease burden, there is a disproportionately weak response to mental illness, characterised by a lack of prioritisation of resources and policies [7]. There is very low spending in mental health, with the bulk of the inadequate financial resources allocated to neuropsychiatric hospitals and less to community-based services regardless of evidence-based recommendations [8]. Community-based mental health services, which include PHC, specialist mental health services in the PHC setting, and non-health sector sheltered employment, residential, and care [9], are underfunded and understaffed resulting in a weak mental healthcare system. Therefore, providing evidence to address the neglected mental healthcare management of mental illness at Primary Health Care (PHC) or community level will go a long way in fulfilling the aim of universal health coverage under the Sustainable Development Goals (SDGs).

Community-based services, which include PHC, are considered the most effective approach to addressing mental illness and providing mental health care [9]. The WHO Mental Health Action Plan 2013–2020 recommends the integration of mental health and social care services as a strategy to improve mental health outcomes [9]. It recognises the importance of mental health in the sustainable development of all countries and supports the strengthening of PHC as part of the universal health coverage drive [9]. In addition, the Lancet Commission on Global Mental Health and Sustainable Development acknowledges that mental health services should be a vital component of universal health coverage, and should be prioritised like other health challenges such as HIV/AIDS [10].

Despite evidence supporting investment in community services, mental health services are underfunded and under-prioritised in most low- and middle-income countries, with the bulk of the scarce resources targeting specialised mental healthcare hospitals and neglecting community services [9]. Reasons for lack of community mental health prioritisation are multifaceted and range from lack of psychiatrists at PHC setting, an understaffed health workforce, ill-equipped and trained staff, lack of policies and political will, and lack of financial investment [11]. To address these challenges, there is a need to provide evidence to strengthen the provision of mental health services at the community level. In providing an overview of the quantity, quality and gaps in the evidence to strengthen the provision of mental health services at the PHC setting, a systematic evidence mapping process [12] was undertaken. This project was taken as part of a request from the Clinical Unit, Sedibeng District of Gauteng, to inform best practices and decision making regarding mental health services and systems in the Gauteng province, other provinces in South Africa, as well as a future research agenda.

## Why an evidence map on strategies to strengthen the provision of mental health care services at the PHC setting is important for South Africa

In 2015, in the Gauteng Province, South Africa, over 1400 people with severe mental illness were abruptly transferred from institutional care at Life Esidimeni hospitals to alternative care facilities, most of which were community-based, and reliant on district health services to provide health care. Within a year of being transferred, 8.3% (119 people) died, despite the urgent rehospitalisation of many patients [13]. The Life Esidimeni tragedy highlights the desperate need to provide appropriate health care at the community level.

The Constitution of the Republic of South Africa assures people with mental illness of their basic human rights, including the right to health care. The Mental Health Care Act No.17 of 2002 (‘the Act') enshrines the right of mental health care users to receive care, treatment and rehabilitation (CTR) close to their homes and in the least restrictive environment [14]. The CTR must enable the user to achieve their full potential, facilitate their reintegration into the community (Section 8(1)), and be in proportion to the user's mental health status (Section 8(2)). Thus, hospital-based care must be justified, and community-based mental health care is endorsed.

The poor resourcing of mental health care, inequity between provinces, lack of routinely collected data, and ongoing reliance on stand-alone mental hospitals, led to the publication of the National Mental Health Policy Framework and Strategic Plan 2013–2020 [15]. This plan aims to ensure that quality mental healthcare services are accessible, equitable, comprehensive and integrated at all levels of the health system, including PHC, in line with the WHO Mental Health Action Plan 2013–202. This includes a model for community-based services with human resources to provide specialist care at the district level [16–17].

## Materials and methods

Evidence maps are an evidence synthesis methodology that systematically selects, identifies and organise a body of knowledge, to provide a high-level picture of the size and nature of the available evidence to inform decision making and practice [12]. The body of knowledge is usually systematic reviews and their inclusion is one of the characteristics of generating Evidence Maps. The Evidence Map (EM) is usually in a user-friendly visual format that makes it easy for the user to interpret the evidence.

### Search strategy and inclusion/exclusion criteria

The EM followed a comprehensive and systematic search process that facilitated the identification of the strategies that might improve the provision of mental health services at the PHC setting. The search strategy and screening followed the systematic review methodology.

### Scoping and search terms

We conducted an initial scoping of the literature to clarify our question, develop a strategy for the EM and to confirm that there was relevant literature available. As part of the scoping, we conducted targeted searches, using limited words, in PubMed and PsycINFO, and out of 141-screened abstracts, only one review that looked at care for patients with serious mental illness treated in primary care setting was relevant for the review question. This review was conducted by the Norwegian Knowledge Centre for the Health Services in 2007 [18] and focused on collaborative care and it derived some of its findings from another review by Craven and Bland [19]. Our initial scoping search had not picked up this second study of Craven and Bland.

For the main search, we expanded our initial scoping strategy search in four ways. Firstly, we decided to copy the Norwegian study's [18] search strategy as far as possible, as it contained a full list of mental illnesses and focused on primary health care. Secondly, through a conversation with a mental health expert in South Africa, we identified a systematic review of psychosocial rehabilitation and included their search terms in our strategy [20]. Thirdly, instead of limiting our review process to serious mental illness, we decided to expand and include all mental illnesses treated at the PHC level. Lastly, we identified the PubMed MESH terms used to index the Craven and Bland review [19] and added these to our search strategy.

### Inclusion and exclusion criteria

Based on the objectives, we included articles that met the following parameters:

- Population: Reviews that synthesise the evidence of the effect for mental health interventions for adult clients (18 years and older) with a mental illness.
- Intervention: Any strategy to strengthen the provision of mental health care at the PHC setting. The strategies could be concerned with general primary care, specialist psychiatric care or any psychiatric treatment offered to those with a mental illness.
- Comparator: We presumed each review would have decided on what comparators are acceptable in the studies to be included in their review. Therefore, the comparator was not part of our inclusion criteria.
- Outcome: Given that our topic covered a broad range of severe mental illnesses, the type of outcomes ranged from service level indicators (e.g. hospital admissions, numbers attending care, cost-effectiveness), the process of care indicators (e.g. adherence, attendance at clinic appointments), to the patient or family-level outcomes. Patient or family level outcomes included measures of function, quality of life, improvement in symptoms, as well as patients' acceptability of the intervention.
- Setting: Interventions implemented within PHC across high, middle or low-income country settings.
- Study type: Systematic reviews

We excluded systematic reviews focusing on children and adolescents and where the intervention or strategy was not at the PHC setting, or where the focus of the review was the efficacy of a specific treatment (e.g. a drug or a therapy), see Table 1.

**Table 1:** Strategies to strengthen provision of mental healthcare.

Strategy	Description
Specialised community-based services	These services manage mental illness (chronic illness) through formalized links between primary and specialized care. These interventions are run by specialists (psychiatrists, psychologists, occupational therapists) but located in the community/ PHC setting.
Integration of Care/Collaborative interventions	These are intensified and structured systems of collaboration between health professionals with specialised psychiatric expertise and primary care health providers to proactively manage mental illness as a chronic disease This any model of care where different cadres of health workers collaborate, or where physical and mental health care is provided in an integrated way.
Task-shifting/Sharing approaches	This is where less skilled / trained cadres take on tasks normally carried out by more highly trained staff. This approach involves the rational redistribution of specific, where appropriate, from highly qualified mental health workers to health workers with shorter training and fewer qualifications in order to make more efficient use of the available human resources for health.
E-health interventions	These are any interventions that involve health services and information being delivered or enhanced through the Internet and related technologies. The rationale is that such technologies will relieve the workload of PHC staff
Group therapy vs. individual therapy	Group therapy allows one health care worker to support several patients at once and this could potentially relieve the workload of PHC staff.
Strategies that empower families, carers and patients	Enlisting families, carers and patients in the treatment process may improve outcomes, and relieve PHC staff. Some of the strategies that empower families, carers and patients are community residential or day centres, self-help interventions, support groups, vocational interventions, healthy lifestyle interventions, counselling and addressing caregiver burden, psychoeducation, and financial incentives.
Psychotherapy & psychosocial interventions vs./in combination with pharmacotherapy	The balance between psychosocial and pharmacotherapy has implications for human resource and costs at PHC level as well as patient outcomes. A combination of psychotherapy with pharmacotherapy can be effective and allows a multidisciplinary approach to managing mental ill patients. Specialist mental health professionals or non-health sector, community-based organisations can deliver these interventions.
Early detection and preventative strategies	Early detection, prevention and screening strategies all have implications for patient outcomes as well as cost implications. This include when PHC workers screening patients for mental illness and refer then to appropriate services immediately.
Systemic strategies that may change provider behaviour and strengthen the quality of care	Any strategies that may strengthen adherence to clinical guidelines or improve data collection of mental illness. These include continuous professional development, capacity building courses and training of PHC workers on mental health.

### Searching for articles

Our main search was guided by a protocol registered on PROSPERO (CRD42018093161) and reported according to the PRISMA guidelines (S1 Table). Two independent reviewers (WM and JG) searched the following databases: PubMed (via the PubMed/MEDLINE interface using the "PICO" option), Cochrane Library (via The Cochrane Library using MeSH terms and qualifiers), PsycINFO (via the EBSCO interface using keywords), CRD, TRIP, PDQ-Evidence, and Joanna Briggs Institute Database of Systematic Reviews. The search terms (Mental Disorders OR Anxiety Disorders OR Panic Disorder OR Severe mental illness OR Serious mental illness) AND (Primary care for mental health OR integrated primary care for mental health OR mental health primary care OR primary health care OR primary medical care) AND (Systematic review OR comprehensive review OR literature review OR critical review OR integrative review OR review of evidence) were used for each database (S1 File). The last online database searches were on the 21 of October 2018. Additional relevant systematic reviews were identified through citation and reference lists searching for included reviews.

### Article screening

All records from the main search were collated into EndNote software. Duplicates were removed in EndNote and the remaining records were exported to Covidence software, where further duplicates and the screening of titles, abstracts and full-text were performed. The screening was guided by the inclusion criteria described earlier.

### Screening process

All the records were screened in duplicate in Covidence software [21]. Eight reviewers (WM, JG, LR, CR, DC, WO, HM, and TF) were involved in titles and abstracts screening. Before the screening, all eight reviewers screened about 5% (n = 100) of the records on titles and abstracts as training as well as to set a consistency screening pattern. Disagreements during this training were resolved through discussions among the team. The duplicate full-text screening was conducted by six reviewers (WM, DC, CR, JG, LR, ZM), with disagreements resolved through team discussions. After the full-text, screening, included articles were exported to Eppi Reviewer 4 [22] for quality assessment and data extraction.

### Study quality assessment

Identifying a critical appraisal tool that assesses the quality of both effectiveness and qualitative reviews and offers consistent assessment across both reviews, was a challenge. After reviewing existing critical appraisal tools such as the AMSTAR-2 and Joanna Briggs Institute Critical Appraisal Checklist for Systematic Reviews and Research Syntheses, and consultations with evidence synthesis experts and methodologists, the team opted to amend the AMSTAR-2 [23] tool to suit our purpose. The team examined the 16 questions on the AMSTAR-2 tool and ranked them on perceived importance and relevance to the systematic review process, from 1 to 16 (with 1 the most important). Thereafter, the team selected the first 10 ranked questions, modified the wording to suit our process and coded them in Eppi Reviewer 4 (S2 Table). The tool scored each question as ‘yes' (1 point) or ‘no' (0 points). As a way of training as well as to set a consistency assessment pattern, duplicate quality assessment was done on approximately 10% (n = 33) of the included reviews and clarifications on assessing the different types of reviews were made in team discussions that happened during this process. Six reviewers (WM, DC, CR, JG, LR, and ZM) independently conducted quality assessments of the remaining articles. Articles were scored as follows: low quality (0–2 points), moderate quality (3–6 points) and high quality (7–10 points).

### Categorising strategies to strengthen PHC mental healthcare

Identifying and categorising the strategies happened through an iterative process as reviewers screened titles, abstracts and full-texts. Groups of reviews were created in EndNote according to the identified strategies. Through discussions, the team resolved differences and issues arising from categorising the strategies. After a series of team meetings, the following nine PHC strategies were identified (Table 1).

### Data extraction strategy

A data extraction tool (S3 Table) was coded in Eppi Reviewer 4 and used to extract relevant data. Before coding the data extraction tool in Eppi Reviewer, the tool was piloted through duplicate data extraction on approximately 10% (n = 33) of the included articles to ensure quality and consistency. Disagreements and clarifications were discussed and resolved during three meetings. More detail and modification of the data extraction tool was done after each meeting until the team were satisfied and confident in carrying out the data extraction.

Thereafter, all six reviewers (WM, DC, CR, JG, LR, and ZM) extracted data independently on the articles allocated to them via Eppi Reviewer 4. The following data categories were extracted from each article (see Table 2).

**Table 2:** Data extracted categories.

Data category	Description
Type of review	Effectiveness review with meta-analysis/narrative synthesis; qualitative evidence synthesis; mixed method review; realist review; narrative synthesis
Continent*	sub-Saharan Africa; Middle East—and North Africa; Latin America and Caribbean; Europe and Central Asia; South Asia; East Asia and Pacific; North America
Region*	LIC; LMIC; UMIC; HIC; all regions
If the review included a South African study	Yes or no
Population (and condition severity?)	Patients with serious or mild mental illness or both
Strategies to strengthen PHC mental healthcare	E-health; specialised community-based services; task-shifting/sharing; integration of care/collaborative approaches; strategies that empower families, carers and patients; group therapy vs. individual therapy; early detection and preventative strategies; psychotherapy and psychosocial interventions vs./in combination with pharmacotherapy; systemic strategies that may change provider behaviour and strengthen the quality of care
Outcomes	Hospital admissions; adherence in care and treatment; retention in care; staff knowledge/skills; psychiatric/clinical symptoms; functional/quality of life; cost-effective; family/societal; feasibility, acceptability, safety and usability; adverse events
Quality score of the review	Low, moderate or high

*World Bank Regions and Income Groups as of June 2018

The designing and development of the evidence map did not report on the direction of impact of the outcomes but it describes the amount and quality of evidence linking each strategy with outcomes as well as indicates the areas with knowledge gaps. To understand what each strategy entails, a narrative synthesis is provided below.

### Evidence mapping

The extracted data was compiled into a JSON file in EPPI Reviewer 4 and exported into an evidence map wizard, which created the EM with linkages between each strategy and outcome types. Each strategy (rows) were mapped on to different outcomes (columns) (see Table 3). Since some articles had more than one intervention and one outcome, it was applicable for articles to be mapped to more than one cell within the Evidence Map. The number and colour of the blocks in the Evidence Map indicate the amount and quality of evidence of the link between a strategy and an outcome.

**Table 3:** Results matrix.

Strategies that might strengthen provision of mental health at PHC		Specialised community-based services	Integration of Care/Collaborative Interventions	Task-shifting/Sharing approaches	E-health Interventions	Group therapy vs. individual therapy	Strategies that empower families, carers and patients	Psychotherapy and psychosocial interventions vs./in combination with pharmacotherapy	Early detection and preventative strategies	Systemic strategies that may change provider behaviour and strengthen the quality of care
**Population**	**Serious mental illness**	21*	56	10	33	9	82	26	22	17
	**Mild/moderate mental illness**	4	37	11	29	9	45	25	22	8
**Type of Review**	**Effectiveness review with meta-analysis**	12	27	5	20	8	44	17	16	4
	**Effectiveness review with narrative synthesis**	8*	23	4	26	2	43	12	13	10
	**Qualitative evidence synthesis**	0	2	1	0	1	4	1	1	0
	**Mixed method review**	0	0	0	1	0	0	0	0	1
	**Systematic narrative synthesis**	0	7	1	2	0	5	4	1	7
	**Other reviews**	1	1	0	0	0	7	2	1	1
**Quality**	**High quality (7–10)**	16*	34	6	33	10	62	17	16	15
	**Moderate quality (3–6)**	3	19	4	15	1	28	17	13	3
	**Low quality (0–2)**	2	7	1	0	0	11	2	2	3
**World Bank Income Groups**	**LIC**	1	1	1	0	0	1	0	0	1
	**LMIC**	3	4	4	2	0	8	2	0	2
	**UMIC**	3	3	3	7	1	21	2	1	1
	**HIC**	15*	40	7	29	8	45	16	14	9
	**All income groups**	0	2	0	1	1	2	1	1	0
	**Not mentioned**	4	12	3	18	2	48	20	13	11

*The number in each cell represents the systematic reviews reporting on each strategy, quality and World Bank Income group where such interventions were implemented

## Results

### Number, type and geography of included articles

**Fig 1:**
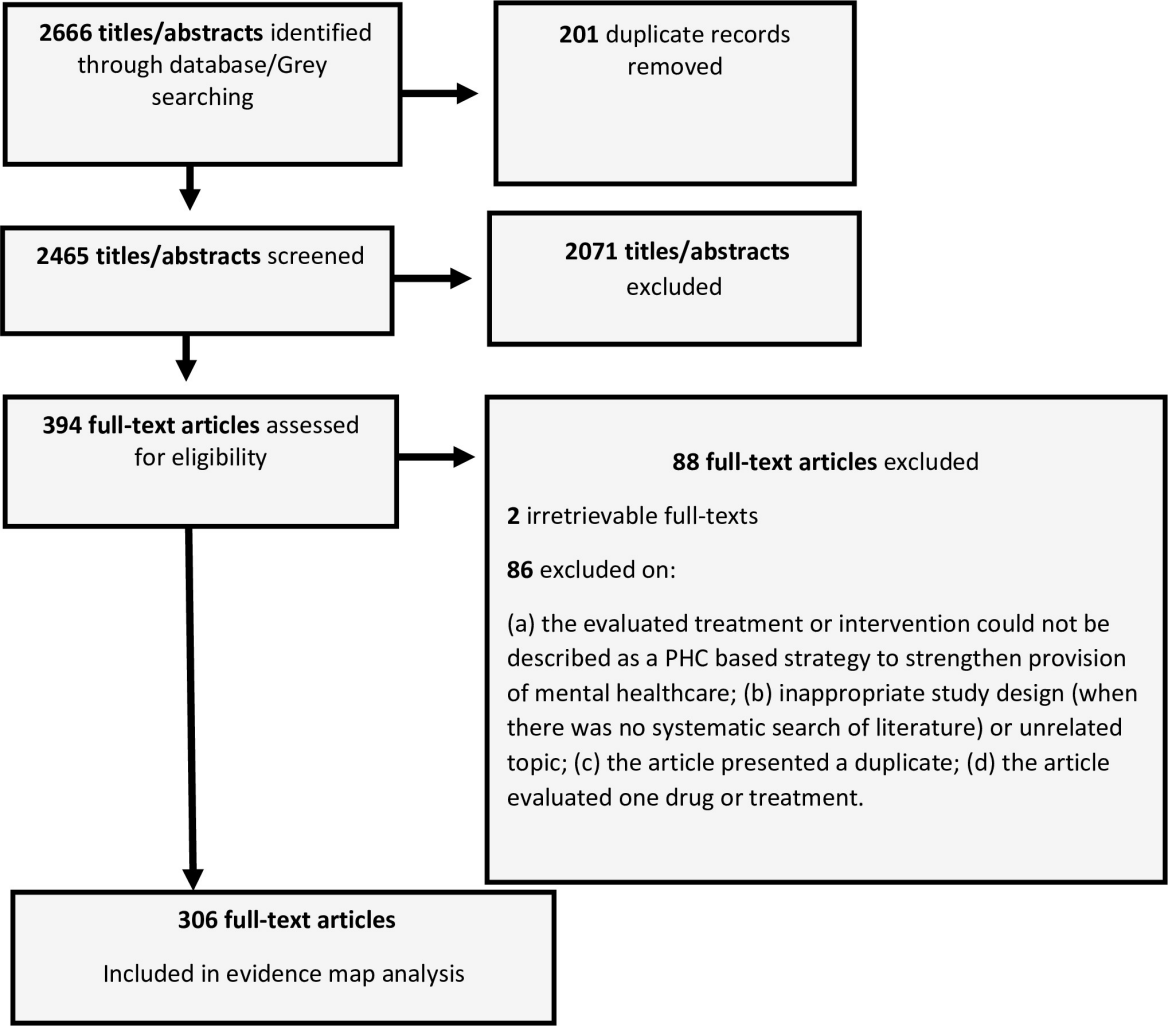
Diagram showing step-by-step results of the systematic search of evidence.

A PRISMA flow chart (Fig 1) shows the step by step systematic searching of evidence included in the EM. Electronic database and reference searching yielded 2666 unique results. Duplicates (201) were excluded. Title and abstract screening identified 394 articles for full-text screening, with 2 of those articles irretrievable. Of the 392 articles that went for full-text screening, 86 were excluded for the following reasons: (a) the evaluated treatment or intervention could not be described as a PHC based strategy to strengthen provision of mental healthcare; (b) inappropriate study design (when there was no systematic search of literature) or unrelated topic; (c) the article presented a duplicate; (d) the article evaluated one drug or treatment. Ultimately, 306 articles were included in the final EM.

Of the 306 included systematic reviews, 9 were published before 2000, 29 between 2000 and 2005 and the remaining 268 articles were published after 2005. Most of the included articles were effectiveness reviews with a meta-analysis and of high quality. The included articles span a range of World Bank regions and income groups. Most of the reviews covered the following areas; North America, Europe and Central Asia, East Asia and Pacific, with relatively few articles from sub-Saharan Africa, Middle East and North Africa, and Latin America and Caribbean. High-income countries including United States of America (US), United Kingdom (UK) and Australia, accounted for most of the included articles (Table 3). The results matrix (Table 3) shows the nine identified strategies that might strengthen the provision of mental health at PHC (as columns) and the types of reviews included in the EM. In addition, the results matrix indicate the World Bank Income Group of where the reviews were conducted, the quality of the review, and the type of patients, which were studied.

### Types of strategies that promote the provision of mental health care at the PHC setting

Almost a third (n = 100) of the articles described strategies that empower families, carers and patients, as a way of strengthening the provision of mental healthcare. Enlisting families, carers and patients are reported as a way that may improve outcomes, and relieve PHC staff. Some of the reported strategies that empower families, carers and patients are community residential or day centres, self-help interventions, support groups, vocational interventions, healthy lifestyle interventions, counselling and addressing caregiver burden, psychoeducation, and financial incentives.

Integration of care or collaborative interventions, such as care models, case management, consultation-liaison, inter-professional collaboration and shared patient-doctor decision-making, where the second (n = 58) most described strategy. Integration of care or collaborative interventions are models of care where different cadres of healthcare workers collaborate, or where physical and mental healthcare are provided in an integrated were. The third (n = 49) most described strategy is eHealth interventions, which are various healthcare practices that are supported by electronic processes and social media communication technologies. The rationale behind eHealth is that such technologies will relieve some of the workloads of PHC staff and some of these technologies are telemedicine, web-based therapy and mHealth.

The balance between psychosocial and pharmacotherapy has implications for human resources and costs at the PHC level as well as patient outcomes. Psychotherapy and psychosocial interventions in combination with pharmacotherapy, were the fourth (n = 36) most reported strategy that might strengthen the provision of mental healthcare. Early detection and preventative strategies were the fifth (n = 31) most described strategy. Early detection, prevention and screening strategies all have implications for patient outcomes as well as cost implications, and these strategies involve identifying high-risk individuals, screening tools for early detection and interventions for prevention.

Specialised community-based services and systemic strategies that may change provider behaviour and strengthen the quality of care are both the sixth (n = 21) most described strategies. Specialised community-based services are interventions run by specialists but located in the community, such as community mental health teams or Assertiveness Community Teams (ACT), and day hospitals or clinics. Systemic strategies (n = 21) that may change provider behaviour and strengthen the quality of care, can be monitoring frameworks for process or patient outcomes, improved data collection on mental illness at PHC, interventions to strengthen adherence to clinical guidelines or financial incentive frameworks for healthcare providers.

Lastly, task shifting or sharing approaches (n = 13) and group therapy versus individual therapy (n = 11) are the least reported strategies. Task-shifting or sharing approaches are where less skilled or trained cadre take on tasks normally carried out by more highly trained staff, such as task shifting for mental health needs or physical needs of the mentally ill patients. Group therapy allows one healthcare worker to support several patients at once and this could potentially relieve the workload of PHC staff. Group therapy can be cognitive behavioural, vocational activities or occupational therapy.

### Outcomes

The 306 identified reviews report on several different outcomes impacted by the strategies mentioned above. Outcomes were grouped into 10 categories as follows: hospital admissions; adherence in care and treatment or retention in care; staff knowledge or skills; psychiatric or clinical symptoms; functional or quality of life; cost-effective; family or societal; feasibility, acceptability, safety and usability; and others. An additional outcome, no studies met the inclusion criteria, was created to cater to reviews that did not find studies to include.

About 80% (n = 245) of the reviews assessing all of the nine strategies that might strengthen the provision of mental healthcare services at PHC level, reported the following outcomes: improve the psychiatric or clinical symptoms of patients, reduce hospital admissions, improve adherence in care and treatment or retention in care, improve functional or quality of life of patients, cost-effective, and patients, carers and healthcare workers find them feasible, acceptable, safe and useable.

A total of 34 reviews assessing integration of care or collaborative interventions, strategies that empower families, carers and patients, psychotherapy and psychosocial interventions in combination with pharmacotherapy, early detection and preventative strategies and systemic strategies that may change provider behaviour and strengthen the quality of care, all indicated impact on family and societal views and perceptions towards mental health. In addition, four reviews evaluating specialised community-based services, integration of care or collaborative interventions and early detection and preventative strategies, reported that these three strategies reduce hospital or clinic waiting times and shorten the scheduling of appointments process.

Twenty-two reviews on strategies that empower families, carers and patients, early detection and preventative interventions, task-shifting approaches and integration of care, show a varying impact on improving healthcare staff knowledge or skills on mental health. Fourteen reviews evaluating E-health, specialised community-based services, integration of care interventions and early detection and preventative interventions, reported mixed impact on other outcomes, such as returning to formal employment, improving budgeting, community reintegration and access to social services such as housing. Lastly, seven reviews did not report on any outcomes because they did not identify studies that met their inclusion criteria.

### Evidence map

**Fig 2:**
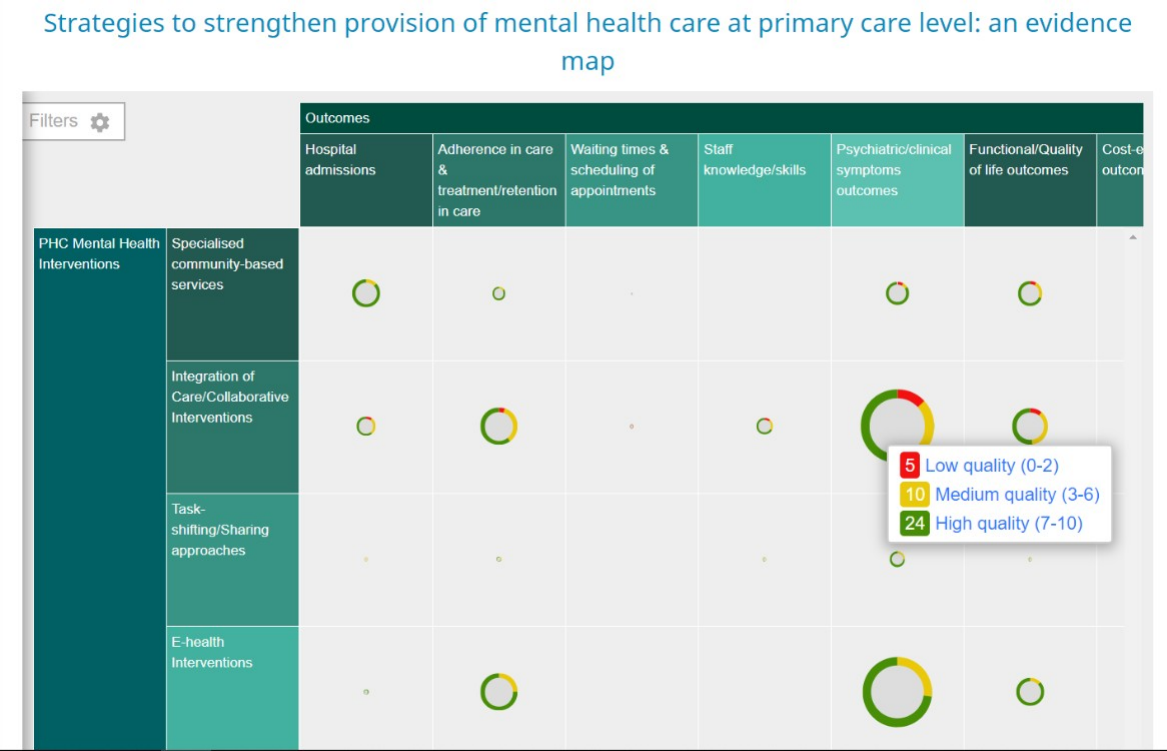
Evidence map of strategies to strengthen provision of mental health care at PHC level.

The evidence map of the identified strategies to strengthen the provision of mental healthcare at the PHC setting is represented in Fig 2. The strategies vs outcome relations derived from the 306 included reviews are mapped out alongside each other. The EM shows the potential impact that each strategy might have on several outcomes, for example, there are 39 reviews on the integration of care/collaborative interventions that indicate a positive impact on patient psychiatric/clinical outcomes. The map further indicates the grading of the evidence of each included review.

There is much evidence on strategies that empower families, carers and patients: integration of care/collaborative interventions, and e-health interventions. These strategies have been shown to have some kind of impact on almost all the reported outcomes. Evidence on specialised community-based services, task-shifting/sharing approaches and group therapy versus individual therapy is not much.

## Discussion

In as much as hospital-based mental health care have been justified for years [24], the effect of strategies to strengthen the provision of primary health or community-based mental health care is supported by various levels of evidence. Some of the identified strategies on the EM have been recommended and endorsed for implementation by the WHO Mental Health Action Plan 2013–2020 [25]. PHC mental health has been portrayed as a potential panacea to tackling the ever-increasing burden of mental illness and the identified evidence of the strategies indicate some positive impact in strengthening the provision of PHC mental health care.

The iterative process employed in the screening, identifying of included systematic reviews, quality assessment and identifying of the nine strategies that have been reported in the evidence map, was robust as each activity involved three or more reviewers. However, it should be noted that the identified strategies are based on how the reviewers understood and agreed to the group and name them. For example, understanding the integration of care/collaborative approaches might differ with context and content of how the implementation was conducted and reported. In addition, the reported outcomes as indicated in the EM were grouped in some instances according to the understanding of the reviewers based on how they defined and explained in the reviews.

Although our evidence points to some positive impact of various strategies in strengthening provision of PHC mental health care, this evidence map does not explicitly show the magnitude of impact towards reviewed outcome categories. Therefore, the implementation of any of the identified strategies needs to be treated with an understanding that this evidence map is just showing the amount and quality of evidence supporting each strategy. For a full understanding of the magnitude of the impact of each of the identified nine strategies towards the reported outcomes, the team will also conduct a specific overview of reviews. In conducting the specific overview of reviews, additional specific searches of literature on each of the nine strategies will be done because there is a likelihood that additional systematic reviews and studies not yet included in systematic reviews will be identified.

As there is a drive to adopt and implement strong PHC health systems for mental health in low- and middle-income countries [25], our EM pose an opportunity to open and stimulate discussions on policy-informed formulation and implementation of strategies to improve the provision of mental health services at the PHC. In the context of this map and the amount of evidence shown, there might be justification for the adoption of some or all of the identified strategies to strengthen the provision of PHC mental health care. However, most of the evidence in the map came from high-income countries, such as USA, UK and Australia, therefore, policy/decision-makers, researchers and other different stakeholders need to understand that besides evidence, social, political and economic contexts greatly influence the adoption and implementation processes. In addition, it may be worth noting that some recent research conducted in LMICs have not yet been captured in systematic reviews. Therefore, this evidence map should be viewed only as one tool of many others, which can be used to strengthen the provision of mental health care at the PHC level.

## Conclusion

This paper presents a map of evidence on the strategies that can strengthen the provision of mental health care at PHC level. Using systematic review methodology and critical appraisal of the included reviews, this paper provides a comprehensive overview of strategies that can be implemented in PHC to strengthen mental health management. In addition, the map also portrays the amount, quality and region of where the evidence came from.

There are various levels of evidence on the nine different strategies, which have shown a potentially positive impact on several outcomes ranging from psychiatric/clinical symptoms of patients, hospital admissions, functional/quality of life, and health worker knowledge and skills. We believe that our EM is an important contribution to the ongoing discussions of what, why and how PHC mental health care can be strengthened as part of a comprehensive health systems drive in light of the 2030 SDGs.

To facilitate wider knowledge translation and stimulate discussions around strengthening mental health care at PHC level, the evidence map is already being used to engage with clinicians, researchers, policy/decision-makers and mental health stakeholders from different provinces of South Africa and sub-Saharan African countries. These engagements are facilitating a broader understanding of how to take forward this evidence map in a way that is useful and meaningful to clinicians, decision-makers, mental health stakeholders, advocacy groups and the public at large. Through these interactions, requests for specific evidence (overview of reviews) on some of the nine strategies have been made as the drive to strengthen the provision of mental health care at PHC level gains momentum.

## Supporting information

S1 File Search strategy. (PDF)

S1 Table PRISMA checklist. (PDF)

S2 Table Quality assessment tool. (PDF)

S3 Table Data extraction form. (PDF)
