# Supplementary material for: Strategies to strengthen the provision of mental health care at the primary care setting: An Evidence Map
Source: PLoS One. 2019 Sep 6;14(9):e0222162. doi: 10.1371/journal.pone.0222162 (PMC6731011; doi:10.1371/journal.pone.0222162)
Supplement: S1 File — (PDF) [file pone.0222162.s001.pdf]

S1 File. Search Strategy

- Population terms: *Mental Disorders OR Anxiety Disorders OR Panic Disorder OR Phobic Disorders OR Eating Disorders OR Anorexia Nervosa OR Bulimia Nervosa OR Mood Disorders OR Affective Disorders, Psychotic OR Bipolar Disorder OR Depressive Disorder OR Depressive Disorder, Major OR Personality Disorders OR Antisocial Personality Disorder OR Borderline Personality Disorder OR Compulsive Personality Disorder OR Dependent Personality Disorder OR Histrionic Personality Disorder OR Paranoid Personality Disorder OR Passive-Aggressive Personality Disorder OR Schizoid Personality Disorder OR Schizotypal Personality Disorder OR Paranoid Disorders OR Psychotic Disorders OR Schizophrenia OR Schizophrenia, Catatonic OR Schizophrenia, Disorganized OR Schizophrenia, Paranoid OR Severe mental illness OR Serious mental illness*

AND

- Intervention terms: *Primary care for mental health OR integrated primary care for mental health OR mental health primary care OR primary health care OR primary medical care OR psychosocial rehabilitation OR primary health care rehabilitation OR occupational therapy OR psychotherapy OR Patient care team OR patient-centred care OR Comprehensive health care OR Psychology, medical OR Delivery of health care, integrated OR cooperative behaviour OR family practice OR interprofessional relations OR psychiatric nursing OR Interdisciplinary communication OR patient care planning OR psychotropic drugs, therapeutic use OR Combined modality therapy OR collaborative mental health care OR Psychoeducation OR adherence OR cognitive rehabilitation OR collaborative care OR family interventions OR community psychiatry OR community rehabilitation OR telemedicine OR e-health OR mobile phones OR self-help OR task-shifting OR family approaches*

AND

- Study design: *Systematic review OR comprehensive review OR literature review OR critical review OR integrative review OR 'review of evidence' OR Benchmarking methods OR Evidence-Based Medicine OR Analysis of the Evidence Base OR qualitative evidence synthesis OR Evidence qualitative synthesis*
