# Supplementary material for: Strategies to strengthen the provision of mental health care at the primary care setting: An Evidence Map
Source: PLoS One. 2019 Sep 6;14(9):e0222162. doi: 10.1371/journal.pone.0222162 (PMC6731011; doi:10.1371/journal.pone.0222162)
Supplement: S2 Table — (PDF) [file pone.0222162.s003.pdf]

S2 Table. Quality assessment tool

Quality questions	Yes (1 point)	No (0 point)
1. Is the review question clearly and explicitly stated		
2. Did the authors set out their inclusion and exclusion criteria clearly		
3. Did the authors conducted a comprehensive literature search, i.e, all three below<br>a. At least 2 databases<br>b. Provided key words or search strategy<br>c. State publication restrictions		
4. Did the review authors perform study selection in duplicate		
5. Did the authors perform data extraction in duplicate		
6. Did the authors describe the included studies in sufficient detail?<br>a. Population, intervention, outcomes, study design (for quant studies) OR<br>b. Setting, perspective, intervention / exposure, comparison, evaluation (how success was determined)		
7. Were the studies critically appraised?		
8. Was the quality of the included studies accounted for when interpreting the results of the review?		
9. Did the authors discuss any heterogeneity among the included studies? (e.g how differences in context or intervention might affect the synthesis)		
10. Did the authors report no conflict of interests AND sources of funding		
