# Supplementary material for: Strategies to strengthen the provision of mental health care at the primary care setting: An Evidence Map
Source: PLoS One. 2019 Sep 6;14(9):e0222162. doi: 10.1371/journal.pone.0222162 (PMC6731011; doi:10.1371/journal.pone.0222162)
Supplement: S3 Table — (PDF) [file pone.0222162.s004.pdf]

S3 Table. Data Extraction Form

**1. Administrative information**	
1.1 Study title	
1.2 Publication year	
1.3 Author(s)	
1.4 Type of review	☐ Effectiveness review with meta-analysis – *a review that provides effectiveness through pooled effect sizes for either subgroups or overall group*
	☐ Effectiveness review with narrative synthesis – *a review that describes effectiveness without pooling of data*
	☐ Mixed review – quantitative & qualitative studies – *a review of both qualitative and quantitative studies systematically drawn together to present evidence on a particular issue*
	☐ Qualitative evidence synthesis – *a review of systematic syntheses of qualitative evidence which normally reports perceptions, opinions or experiences*
	☐ Realist review – *a review that examines the theories that explains why an intervention works, for whom and in what circumstances*
	☐ Systematic narrative synthesis – *a review that can consider any topic that is not effectiveness or perceptions/experiences*
	☐ Other reviews: ________________<br>*Specify the review*<br>******Possible to select one or more***

Key information to extract from each included systematic review

**2. Region/Population**		
2.1 Regions	☐ sub-Saharan Africa<br>☐ Middle East & North Africa<br>☐ Latin America & Caribbean<br>☐ East Asia & Pacific<br>☐ South Asia<br>☐ Europe & Central Asia<br>☐ North America	☐ LIC<br>☐ LMIC<br>☐ UMIC<br>☐ HIC<br>☐ All<br>**** As per World Bank 2018***<br>****Possible to select one or more***
2.2 South Africa	☐ Yes<br>☐ No	
2.3 Country(ies)<br>*State country and/or city*		
2.4 Target group<br>*State which SMI or which group of condition*	☐ Serious mental illness (schizophrenia, major depression, severe anxiety, bipolar disorder etc)<br>☐ Mild-Moderate illness (depression, anxiety)	
**INTERVENTION EVIDENCE**		

Intervention focus

Note: You can tick more than one if the study reports multiple relevant interventions

☐ **Specialised community-based services**
(interventions run by specialists but located in the community)
- Community mental health teams/ACT/other
- Day hospital/clinic

☐ **Integration of Care/Collaborative interventions**
(any model of care where different cadres of health workers collaborate, or where physical and mental health care is provided in an integrated way)
- Care models
- Case management
- Community interventions
- Consultation liaison
- Interprofessional collaboration
- Shared patient healthcare worker decision-making

☐ **Task-shifting/Sharing approaches**
(where less skilled / trained cadre take on tasks normally carried out by more highly trained staff)
- Task-shifting for mental health needs
- Task-shifting for physical needs of mentally ill

☐ **E-health interventions**
(any intervention that involves internet based technology – the rationale is that such technologies will relieve some of the workload of PHC staff)
- Information/technology
- M-health
- Telemedicine
- Web-based therapy

☐ **Group therapy vs. individual therapy**
(as group therapy allows one health care worker to support several patients at once this could potentially relieve the workload of PHC staff)

☐ **Strategies that empower families, carers and patients**
(enlisting families, carers and patients may improve outcomes, and relieve PHC staff)
- Addressing care-giver burden
- Community residential/day centres
- Counselling
- Healthy lifestyle interventions

Psychosocial interventions
Psychoeducation
Psychotherapy
Self-help interventions
Support groups
Vocational interventions
Financial incentives

☐ **Psychotherapy & psychosocial interventions vs./in combination with pharmacotherapy**
(the balance between psychosocial and pharmacotherapy has implications for human resource and costs at PHC level as well as patient outcomes)

☐ **Early detection and preventative strategies**
(early detection, prevention and screening strategies all have implications for patient outcomes as well as cost implications)
Identifying high-risk individuals
Strategies for prevention
Screening tools for early detection

☐ **Systemic strategies that may change provider behaviour and strengthen the quality of care**
Strategies to strengthen adherence to clinical guidelines
Monitoring framework for process or patient outcomes
Financial incentive framework for providers
Improved data collection on mental illness at PHC

OUTCOMES

Note: You can tick more than one if the study reports multiple relevant outcomes

☐ Hospital admissions
☐ Adherence in care & treatment/retention in care
☐ Staff knowledge/skills
☐ Psychiatric/clinical symptoms outcomes
☐ Functional/Quality of life outcomes
☐ Cost-effective outcomes
☐ Family/societal outcomes
☐ Feasibility, acceptability, safety, & usability
☐ Waiting times & Scheduling of appointments
☐ **Others:** ________________ *Specify them*
☐ No studies met the inclusion criteria

Quality Score

☐ **High quality (7-10)**
☐ **Medium quality (3-6)**
☐ **Low quality (0-2)**
